# Demographic, socio-economic, and cultural factors affecting fertility differentials in Nepal

**DOI:** 10.1186/1471-2393-10-19

**Published:** 2010-04-28

**Authors:** Ramesh Adhikari

**Affiliations:** 1 Geography and Population Department, Mahendra Ratna Campus, Tribhuvan University, Kathmandu, Nepal; 2 Institute for Population and Social Research, Mahidol University, Salaya, Thailand

## Abstract

**Background:**

Traditionally Nepalese society favors high fertility. Children are a symbol of well-being both socially and economically. Although fertility has been decreasing in Nepal since 1981, it is still high compared to many other developing countries. This paper is an attempt to examine the demographic, socio-economic, and cultural factors for fertility differentials in Nepal.

**Methods:**

This paper has used data from the Nepal Demographic and Health Survey (NDHS 2006). The analysis is confined to ever married women of reproductive age (8,644). Both bivariate and multivariate analyses have been performed to describe the fertility differentials. The bivariate analysis (one-way ANOVA) was applied to examine the association between children ever born and women's demographic, socio-economic, and cultural characteristics. Besides bivariate analysis, the net effect of each independent variable on the dependent variable after controlling for the effect of other predictors has also been measured through multivariate analysis (multiple linear regressions).

**Results:**

The mean numbers of children ever born (CEB) among married Nepali women of reproductive age and among women aged 40-49 were three and five children, respectively. There are considerable differentials in the average number of children ever born according to women's demographic, socio-economic, and cultural settings. Regression analysis revealed that age at first marriage, perceived ideal number of children, place of residence, literacy status, religion, mass media exposure, use of family planning methods, household headship, and experience of child death were the most important variables that explained the variance in fertility. Women who considered a higher number of children as ideal (β = 0.03; p < 0.001), those who resided in rural areas (β = 0.02; p < 0.05), Muslim women (β = 0.07; p < 0.001), those who had ever used family planning methods (β = 0.08; p < 0.001), and those who had a child-death experience (β = 0.31; p < 0.001) were more likely to have a higher number of CEB compared to their counterparts. On the other hand, those who married at a later age (β = -0.15; p < 0.001), were literate (β = -0.05; p < 0.001), were exposed to both (radio/TV) mass media (β = -0.05; p < 0.001), were richest (β = -0.12; p < 0.001), and were from female-headed households (β = -0.02; p < 0.05) had a lower number of children ever born than their counterparts.

**Conclusion:**

The average number of children ever born is high among women in Nepal. There are many contributing factors for the high fertility, among which are age at first marriage, perceived ideal number of children, literacy status, mass media exposure, wealth status, and child-death experience by mothers. All of these were strong predictors for CEB. It can be concluded that programs should aim to reduce fertility rates by focusing on these identified factors so that fertility as well as infant and maternal mortality and morbidity will be decreased and the overall well-being of the family maintained and enhanced.

## Background

Traditionally, Nepalese society has favored high fertility. Children are considered a symbol of both social and economic well-being. This is evident from a popular saying that says, "May your progeny fill the hills and mountains." Marriage is early and universal, and it is a viewed as a disgrace for a couple, particularly for the wife, not to have children. High fertility is desired because by producing children, preferably sons, a woman raises her status in the family [1]. Other reasons for high fertility include early and universal marriage as well as desire for sons, for both religious (to perform religious rituals) and economic (immediate economic gains and old age security) reasons [2].

The total fertility (TFR) rate has decreased worldwide [3] just as it has in Nepal, where fertility has declined significantly from 6.3 births per women [4] in 1981 to 3.1 births per women in 2009 [3]. The Nepali government began setting fertility reduction targets as early as 1965, but they were never met. During the Third Plan (1965-70) period, the target was to reduce the estimated crude birth rate (CBR) of 39.1 in 1967 to 38.1 in 1971. During the Fifth Plan (1975-80) period, the newly estimated CBR of 40 was to be reduced to 38 by 1980 [5]. Similarly, the Seventh Plan (1985-90) planned to achieve a TFR of about four children per woman by the end of the plan period [6]. But the demographic information shows that Nepal's TFR was above five until the early1990s [7] and above four even in 2001 [8].

The status of Nepal in many of the major demographic and other indicators is very poor [3,9,10]. However, the TFR decreased about 25 percent (4.1 births per women in 2001 to 3.1 births per women in 2006) between the two surveys [8,10]. In particular, the education of women, female labor force participation, urban residence, household wealth, cultural norms, and overall levels of social development have affected fertility [11-14]. Besides these phenomena, many other factors, such as the internal as well as external displacement of people due to the decade-long political insurgency (1995-2006), may also be fundamental reasons for this change in fertility in Nepal. In addition, there has been a tremendous outflow of migrants seeking work in foreign countries, especially in Persian Gulf and Southeast Asian countries. Furthermore, exposure to modern means of communications, increase in family planning awareness, and easy access to modern family planning may also lower the TFR [10].

Fertility is one of the three principal components of population dynamics that determine the size and structure of the population of a country [15]. Differentials in fertility behavior and fertility levels in different areas and among population strata or characteristics have been among the most pervasive findings in demography [16]. Although Nepal has seen substantial improvements in its reproductive health outcomes, such as in the reduction in maternal and infant mortality, the increase in the contraceptive prevalence rate, and health service utilization among married women [10,17], the total fertility rate is still high compared to that of other developing countries [3]. The underlying cause of high fertility in Nepal needs further investigation and exploration in order to be better understood and appropriately addressed by reproductive health programs. It is essential to identify the risk factors associated with high fertility and to provide services to address those who are at risk. To develop effective strategies for fertility control, it is necessary to understand the factors affecting fertility. It is hypothesized that women in vulnerable groups, such as those who got married at an early age, are illiterate, are living in rural areas, are poorest, and have very little knowledge of contraceptives, have high fertility.

This paper is an attempt to examine the fertility differentials among Nepali women. More specifically, it aims to investigate whether demographic, socioeconomic and cultural factors have an impact on fertility in the Nepali context. This paper also aims to guide reproductive health program planners and policymakers to understand various factors influencing fertility in order to assist in the implementation of a reproductive health program that will decrease fertility as well as infant and child morbidity and mortality.

## Methods

### Sources of data

This paper uses data from the Nepal Demographic and Health Survey (NDHS), 2006, consisting of nationally representative sample surveys. The primary purpose of this survey was to furnish policymakers and planners with detailed information on fertility, family planning, infant, child, adult, and maternal mortality, maternal and child health, nutrition, and knowledge of HIV/AIDS and other sexually transmitted infections. The 2006 NDHS was carried out under the aegis of the Population Division of the Ministry of Health and Population. The data used in this study is publicly available.

### Sampling frame and sampling selection

The 2006 NDHS used the sampling frame provided by the list of census enumeration areas with population and household information from the 2001 Population Census. Each of the 75 districts in Nepal is subdivided into Village Development Committees (VDCs), and each VDC into wards. The primary sampling unit (PSU) for the 2006 NDHS is a ward, sub-ward, or group of wards in rural areas, and sub-wards in urban areas. The sample for the survey is based on a two-stage, stratified, nationally representative sample of households. At the first stage of sampling, 260 PSUs (82 in urban areas and 178 in rural areas) were selected using systematic sampling with probability proportional to size. At the second stage of sampling, systematic samples of about 30 households per PSU on average in urban areas and about 36 households per PSU on average in rural areas were selected in all the regions. Interviews were completed for 10,793 women of reproductive age.

### Dependent and independent variables

This paper analyzed the specific measure of fertility; i.e. children ever born (CEB). So the dependent variable used in this paper is CEB. CEB comprises information on the number of all children born alive (lifetime fertility) up to the survey date. Mean number of children ever born to women represents the childbearing experience of a real age cohort and reflects current and past fertility behavior. Finally, CEB does allow for the generalization of data and an understanding that can provide the basis for further analysis [15].

Children ever born, the dependent variable, is treated as an interval scale in both bivariate and multivariate analyses. The children ever born to all women irrespective of the age group (lifetime fertility) and women aged 40-49 (completed fertility) were analyzed separately as a measure of fertility. The demographic, socioeconomic, and cultural variables used as independent variables are: age group of respondents, age at first marriage, place of residence, literacy status, occupation, religion, mass media exposure, wealth status, knowledge, and previous use of family planning methods, household headship, and child-death experience by mothers. Many of the independent variables were categorically variable. For example, the variable perceived ideal number of children refers to respondents' perceptions concerning the ideal number of children they would like to have. This variable is categorized into two categories (up to two children and three or more children) for bivariate analyses and is used as an interval scale for multivariate analysis. Similarly, the variable "religion" measures respondents' religious affiliation. Those respondents who are affiliated with Hindu religion are considered as Hindu, those affiliated with Islam are considered as Muslim, and the respondents affiliated with other religions are categorized as "other religion." Regarding knowledge about family planning methods, almost all respondents (99.5%) had heard of at least one family planning (FP) method. So, the "knowledge of FP" variable is measured by scoring the knowledge of each method. This variable is classified into two categories. The average number of methods heard was taken as a guide for making these two categories. Scores higher than average (average score = 7.1) were classified as "higher" and scores lower than average were classified as "lower" level of knowledge.

Radio and TV are considered as routes of access to health information in this study. A composite index was made from these variables. Then the variable was categorized into three categories. If the respondent was not exposed to any media, the classification was treated as "no exposure." The other categories were "exposed to one media (either radio or TV)" and "exposed to both media (radio and TV)." The categories of the other independent variables were also created in the same way, based on the literature as well as their frequency distribution.

### Methods of analysis

The analysis is confined to women who were ever married (N = 8644). Data were weighted to represent the structure of Nepali population using weighting factors provided with the NDHS. Initially, univariate or descriptive analysis was used to describe the percentage and number of respondents according to sociodemographic characteristics. Both bivariate and multivariate analyses were performed to show the fertility differentials. The dependent variable (CEB) is in continuous scale. We analyzed the mean number of children ever born and other independent variables using one-way ANOVA to examine the association between children ever born and women's demographic, socioeconomic, and cultural characteristics. Furthermore, the net effect of each predictor variable on the dependent variable after controlling for the effect of other predictors was also measured via multivariate analysis (multiple linear regressions). Analysis was performed separately for all women and for women aged 40-49 in both bivariate and multivariate analyses. Before using multiple linear regressions, the correlation matrix has been applied to discover the degree and direction of the relationship between each pair of independent variable and dependent variable. The results demonstrated that there was no multi-colinearity (r > 0.6) between each pair of independent variables and dependent variable. So all the variables included in the bivariate analysis were also included in multivariate linear regression. The Statistical Package for Social Science (SPSS 11.5 for window) software was used to analyze the data.

## Results

### Background characteristics of women

More than a quarter of the ever married women (28%) were youth aged 15-24 and slightly more than a fifth (23%) were aged 40-49. A considerable proportion of the women (33%) were married before the age of 16. A majority of the women lived in rural areas (85%), and most women were illiterate (63%). Similarly, only about one out of ten women (12%) were engaged in non-agricultural sectors. An overwhelmingly large majority of the women were Hindu (86%). Likewise, a large percentage of women had been exposed to at least one mass medium (radio or TV). Furthermore, almost two-fifths of the women (39%) fell into the poor or the poorest category. On average, women were aware of 7.1 family planning methods. A majority of the women (58%) had little knowledge (less than average) of family planning methods. It is notable that more than a third of women (34%) had never used any family planning methods. Furthermore, only about a fifth (21%) were from female-headed households. Surprisingly, more than a quarter of the women (28%) had experienced child-death (Table 1).

**Table 1:** Demographic, socioeconomic, and cultural characteristics of women.

Characteristics		Percent	Number
**Age group**	15-24	28.0	2424
	25-39	48.7	4213
	40-49	23.2	2007

**Age at fist marriage**	Less than 16 yrs	32.6	2814
	16 or more yrs	67.4	5827

**Perceived ideal Number of children**	Up to 2 children	32.6	2814
	3 or more children	67.4	5827

**Place of residence**	Urban	14.8	1280
	Rural	85.2	7363

**Ecological Zones**	Mountain	7.1	611
	Hill	41.4	3576
	Terai	51.6	4457

**Literacy status**	Illiterate	62.6	5409
	Literate	37.4	3234

**Occupation**	Not working	16.3	1411
	Agricultural sector	71.9	6213
	Non-agricultural sector	11.8	1020

**Religion**	Hindu	85.5	7392
	Muslim	4.0	347
	Other	10.5	905

**Mass media exposure**	No exposure	8.6	742
	One media (radio or TV) exposure	26.0	2251
	Both (radio & TV) exposure	65.4	5651

**Wealth Status**	Poor/poorest	38.6	3338
	Middle	21.1	1827
	Rich/Richest	40.2	3479

**Knowledge about FP**	Lower	57.7	4990
	Higher	42.3	3653

**Ever used of FP methods**	Never used	33.5	2898
	Ever used	66.5	5746

**Child death experience+**	No	72.2	5634
	Yes	27.8	2175

**Household headship**	Male headed	79.2	6845
	Female headed	20.8	1799

	**Total**	**100.0**	**8644**

Note + information of child death experienced were collected from women who have ever given birth (n = 7809)

### Demographic, socio-economic, and cultural correlates of children ever born

The mean numbers of children ever born among married Nepali women of reproductive age (15-49) and among the married women aged 40-49 were three children and five children, respectively. Several demographic, socioeconomic and cultural variables were correlated with children ever born. The mean numbers of children ever born to women whose age at marriage was less than 16 years, who perceived a higher number of children as ideal, and who resided in mountain areas were significantly higher than their counterparts.

Regarding literacy status, literate women have only half the CEB than do illiterate women (1.9 vs. 3.7 for all; 3.6 vs. 5.2 for women aged 40-49). Furthermore, Muslim women, women who had never been exposed to mass media, and poor/poorest women had significantly higher CEB than their comparison group. Similarly, those women who had less knowledge about family planning methods had significantly more CEB than those who had a higher level of knowledge about FP. In contrast, women who had used family planning methods before had more CEB than those who had never used them. Notably, women who had a child-death experience had almost double the number of CEB than those who did not have such an experience. Furthermore, the mean CEB is significantly higher among women aged 40-49 in male-headed households compared to women in female-headed households (5.0 vs. 4.7) (Table 2).

**Table 2:** Mean number of children ever born to ever married women aged 15-49 and women aged 40-49 by demographic, socioeconomic, and cultural characteristics.

		All women			Women aged 40-49		
		**Mean CEB**	**SD**	**F-Value**	**Mean CEB**	**SD**	**F-Value**

**Age group**	15-24	1.15	1.0	2797.9***	-	-	-
	25-39	3.22	1.7		-	-	-
	40-49	4.96	2.3		4.96	2.3	-

**Age at first marriage**	Less than 16 yrs	3.6	2.3	304.1***	5.3	2.5	30.9***
	16 or more yrs	2.8	2.1		4.7	2.2	

**Perceived ideal Number of children**	Up to 2	2.4	1.7	1744.3***	4.1	2.1	230.3***
	3 or more	4.2	2.3		5.6	2.3	

**Place of residence**	Urban	2.5	1.8	105.2***	3.8	2.0	79.7***
	Rural	3.1	2.2		5.1	2.3	

**Ecological Zones**	Mountain	3.3	2.4		5.5	2.6	
	Hill	2.9	2.1	11.2***	4.7	2.3	9.2***
	Terai	3.1	2.2		5.1	2.3	

**Literacy status**	Illiterate	3.7	2.3	1657.6***	5.2	2.3	99.5***
	Literate	1.9	1.5		3.6	1.7	

**Occupation**	Not working	2.2	1.8		4.2	2.1	
	Agricultural	3.3	2.3	173.4***	5.1	2.3	25.2***
	Non-agricultural	2.6	1.9		4.4	2.4	

**Religion**	Hindu	3.03	2.1		4.89	2.3	
	Muslim	3.52	2.7	8.9***	6.91	2.6	28.5***
	Other	2.97	2.2		4.83	2.4	

**Mass media exposure**	No exposure	3.9	2.5		5.7	2.4	
	One media (radio or TV) exposure	3.5	2.3	168.9***	5.3	2.4	28.3***
	Both (radio & TV) exposure	2.7	2.0		4.7	2.2	

**Wealth Status**	Poor/poorest	3.5	2.4	134.2***	5.6	2.4	64.9***
	Middle	3.1	2.1		5.0	2.2	
	Rich/Richest	2.6	1.8		4.3	2.1	

**Knowledge about FP**	Lower	3.3	2.3	168.5***	5.1	2.4	21.8***
	Higher	2.7	1.9		4.6	2.2	

**Ever used of FP methods**	Never used	2.5	2.4	278.2***	4.7	2.7	12.2***
	Ever used	3.3	2.0		5.1	2.1	

**Experience of child death**	No	2.7	1.5	3452.7***	4.0	1.8	579.6***
	Yes	5.2	2.1		6.1	2.1	

**Household headship**	Male headed	3.1	2.2	2.7	5.0	2.3	3.9*
	Female headed	3.0	2.0		4.7	2.3	

**Total mean**		**3.0**	**2.2**		**5.0**	**2.3**	

**Total N**		**8644**			**2007**		

Note *** Significant at P < 0.00, ** = p < 0.01 and * = p < 0.05; SD = Standard deviation

### Multivariate analysis

Two separate multivariate analyses (one for all women and one for women aged 40-49) were performed. When all these independent variables and the number of children ever born were included in the logistic model, R-square was 0.59 for the all women category and 0.33 for the women aged 40-49 category. This means that the variables included in the models can explain the variation in the number of children ever born of all women by 59 percent (p < 0.001) and 33 percent (p < 0.001) among the women aged 40-49 years (Table 3).

**Table 3:** Coefficient of demographic, socioeconomic, and cultural variables on number of children ever born: Multivariate analysis.

Predictors	All women		Women aged 40-49	
	
	Coefficient	t-value	Coefficient	t-value
**Current Age**	0.502	59.5***	0.093	4.8***

**Age at first marriage**	-0.152	-20.0***	-0.127	-6.5***

**Perceived ideal number of children**	0.026	3.6***	0.038	2.0*

**Place of residence **Urban (ref.)				
Rural	0.020	2.5*	0.048	2.2*

**Ecological zones **Mountain (ref.)				
Hill	-0.028	-1.9	-0.104	-2.7**
Terai	-0.027	-1.8	-0.095	-2.4*

**Literacy status **Illiterate (ref.)				
Literate	-0.048	-5.4***	-0.079	-3.7***

**Occupation **Not working (ref.)				
Agricultural sector	0.009	0.9	0.015	0.52
Non-agricultural sector	-0.015	-1.6	-0.001	-.05

**Religion **Hindu (ref.)				
Muslim	0.066	8.8***	0.146	7.6***
Other	0.001	0.13***	-0.38	-1.9*

**Mass media exposure **No exposure (ref.)				
One media (either radio or TV) exposure	-0.004	-0.29	-0.030	-1.02
Both (radio and TV) exposure	-0.049	-3.68***	-0.061	-1.96*

**Wealth Status **Poor/poorest (ref.)				
Middle	-0.070	-8.5***	-0.102	-4.83***
Rich/Richest	-0.121	-12.3***	-0.153	-6.16***

**Knowledge about FP **Lower (ref.)				
Higher	-0.001	-0.13	0.017	0.80

**Ever used of FP methods **Never used (ref.)				
Ever used	0.081	10.5***	0.107	5.29***

**Household headship **Male headed (ref.)				
Female headed	-0.016	-2.2*	-0.038	-1.9*

**Experience of child death **No (ref.)				
Yes	0.311	38.9***	0.388	19.73***

**N**	8644		2007	

**R-Square**	0.597***		0.325***	

Note *** Significant at P < 0.001; ** = p < 0.01 and * = p < 0.05

The multivariate analysis found that women's age, age at marriage, perceived ideal number of children, place of residence, literacy status, religion, mass media exposure, wealth status, ever use of FP methods, household headship, and child-death experience by mothers to be significant predicators of fertility in both groups (all women and women aged 40-49). The coefficients shown in Table 3 represent the effect of each independent variable on the dependent variable. Results show that a low age at first marriage significantly and negatively affected the number of children ever born at 0.001 significant levels. To be more precise, increase in women's age at first marriage tended to decrease the number of children ever born, while other variables in the model were controlled (β = -0.15 for all women; -0.13 for women aged 40-49). Similarly, the coefficient (β = 0.026 for all women; 0.038 for women aged 40-49) of perceived ideal number of children showed a significant (p < 0.001) positive effect on fertility. Women who ideally considered it better to have more children were indeed likely to a have higher number of children ever born than those who perceived a low number of children as ideal.

Regarding literacy status, literate women tended to have fewer children than illiterate women (p < 0.001). The study also found out that a woman's religion was an important factor in her fertility. For example, Muslim women were likely to have more children (β = 0.066 for all women; β = 0.146 for women aged 40-49) than Hindu women. On the other hand, Buddhist and Christian women were likely to have fewer children than did Hindu women. Furthermore, those women who are exposed to both mass media (radio and TV) were likely (β = -0.049 for all women; -1.96 for women aged 40-49) to have fewer children than those who were not exposed to any media (Table 3).

Our analysis showed that the rich/richest women were likely to have fewer children (β = -0.12 for all women and -0.15 for women aged 40-49) than those who were poorest. Furthermore, our study showed that household headship also affects fertility. Ever married women from female-headed households were likely to have fewer children than did women from male-headed households (β = -0.016 for all women; -0.038 for women aged 40-49). It is notable that women who had a child-death experience were more likely to have more children than women who did not experience child death (β = 0.31 for all women; β = 0.39 for women aged 40-49) (Table 3).

## Discussion

The study found that on average married Nepali women of reproductive age woman give birth three times and women aged 40-49 five times. This high number of children ever born indicates that more family planning and reproductive health programs are needed. All women, regardless of demographic, socioeconomic or cultural status, would benefit if programs focus on reducing fertility.

Considerable differences in CEB according to women's age, age at marriage, perceived ideal number of children, place of residence, literacy status, religion, mass media exposure, wealth status, ever use of FP methods, household headship, and child-death experience by mothers were found in the study. As in other studies, our study also found that older women have a significantly higher number of CEB. It could be that the expected number of children will increase as women's age increases [18]. Similarly, as we hypothesized, those women who married early were likely to a have higher number of children than their counterparts. An increase in the age at first marriage has an adverse effect on high fertility. Early marriage not only marks a woman's entry into a sexual union and the beginning of exposure to childbearing but may also be an important gauge of women's status, since the older the woman is when she marries, the greater the likelihood that she has attended school or been employed, and the greater her chances of having a more equal relationship with her husband [19]. Our finding is similar to many other studies that find that older age at first marriage played an important role in reduction in fertility [20-23]. Furthermore, our study found that women who perceived more children as ideal were likely to have more children ever born than those who perceived a low number of children as ideal. It could be that the ideal number of children is one of the most important predictors in determining women's desire to stop childbearing and one that affects women in terms of their accepting contraceptives and fertility planning [24].

The present study also shows that rural women have higher fertility than urban women. Our study result is similar to many other studies [25-28]. One reason could be that urban women are more likely to use contraceptives than are rural women; therefore, the fertility levels in urban and rural areas tend to be different [29,30]. The other reason could be that people who live in rural areas tend to marry at a younger age than do those in urban areas [31]. Similarly, the higher CEB among women in mountain areas could be because mountain areas are sparsely populated and also less developed overall than other ecological regions. Mountain regions are also remote and inaccessible compared to most Terai (the southern plains) and hill settlements, which are accessible by road. Socioeconomically too, the Terai region is better off. The other reason could be that more than half the urban population lives in the Terai [1].

It is well known that the fertility of a woman is negatively associated with her level of education [32]. We hypothesized that illiterate women are more likely to have a higher number of children than are literate women. This study also showed that illiterate women have almost double the number of CEB than do literate women. Education exposes women to information, empowers women, makes them more likely to be employed outside their home environment, and makes them more aware of their own health and the health of their children-all of which are negatively associated with the number of children a woman will have during her reproductive life. Similarly, educated women are more likely to postpone marriage, have smaller family size, and use contraception than are uneducated women [33]. Other studies also suggest that education has a negative effect on women's cumulative fertility [34-36].

This study found that Muslim women were more likely to have more children than were to Hindu women. It could be that religion has an immense social, economic, and political significance in most societies, and thus plays an important role in sanctioning or promoting acceptance of or creating resistance to family planning [37-40]. Although both Islam and Hinduism are pronatalist religions, these religions differ in regard to their beliefs concerning marriage, reproductive behavior, and fertility control [41]. Our study finding is similar to that of many other studies [42-45].

Throughout the world, mass media have influenced knowledge, attitudes, and behavior regarding the use of contraception [46]. Our study also found that mass media exposure (radio/TV) has an important effect on reproductive behavior. Those women who were exposed to radio/TV had fewer children than those who were not exposed. It could be because radio and television programs and the values they disseminate are transmitted directly into the home, they have the potential to directly affect every member of the household, even those with little or no schooling [47,48]. The role of mass media in changing both patterns of contraceptive use and notions of ideal family size could be another reason for low fertility among those exposed to mass media [49].

We hypothesized that the poorest women would have higher fertility than the richest women. The relation of wealth and fertility can be clearly seen in the study. An inverse relationship was observed between wealth status and fertility, with significantly lower fertility among the richest women compared to fertility among the poorest. This result is the same as for other studies [50,51]. The reason could be that poor people may perceive children as a source of income, thus motivating them to have more children [2]. Another reason could be that the poorest people have less access to education and family planning methods.

In contrast to other research [52-54], our results indicate that those women who had ever used contraception had more children ever born than those who did not use contraception. It could be that women adopt contraception when they reach or exceed the target number of children they would like to have [55].

Another interesting finding of this study was that women from female-headed households had fewer children than women from male-headed households. During the recent period of conflict in Nepal, many people, especially males, have left their villages and have gone to other countries for employment. The low fertility among female-headed households could be due to the extended separation of women from their husbands. Furthermore, almost three out of four women (73%) from female-headed households were themselves the head of their household. Most of these female household heads had high status in their family. Previous research has also shown that women's high social status is positively correlated with their health status [56,57]. A study in Sri Lanka found that women in households headed by females used health services more frequently than those in male-headed households [58]. The possible explanation could be that women who have autonomy in decision making are more likely to have higher level of contraceptive use, which might ameliorate their reproductive behavior risks and result in longer birth intervals and low fertility [59].

A relationship between fertility and mortality can easily be found in our results. Women who had a child-death experience were likely to have a higher number of children than those who had no such experience. As the number of children who died increased, women were exposed to a higher risk of uncontrolled fertility. Casterline suggests that where mortality declines more rapidly, the pace of fertility decline will also be more rapid [60]. Similar results can also be found in many other studies [61-64], which show that child mortality had a significant positive effect on fertility, that is, that an increase in the child mortality rate would significantly increase fertility.

There are some limitations with regard to interpreting the results of this study. Due to the cross sectional design of the study and all the variables analyzed in the regression model, it can only provide evidence of a statistical association between those variables and children ever born and cannot show a cause-effect relationship. The other limitation of the study is, as a measure of fertility, the number of CEB suffers from problems of truncation and censoring as it includes the number of children born up to specific points in women's childbearing years. We should not forget other important errors that could exist in the collection of information regarding the number of children ever born. Women tend to omit some of the children they have given birth to, particularly those living in other households and those who have died, with the result that the proportion children omitted tends to increase with the age of the mother. Another error in the reported number of children ever born arises from the inclusion of stillbirths or late fetal deaths among live-born children [15]. However, there may in fact be few errors in this regard as it is believed that the Nepal Demographic and Health Survey data were good enough to estimate fertility directly [1,10].

## Conclusion

In Nepal, the number of children ever born is high. Many factors contribute to this phenomenon. Among these factors, age at first marriage, perceived ideal number of children, literacy status, mass media exposure, wealth status, and experience of child death are important and strong predictors that affect fertility. Despite the legal restrictions against marrying at a young age, early marriage is common in the country. Therefore, programs should focus on creating awareness of the marriage law and the disadvantages of early marriage and large family size. Similarly, more emphasis needs to be place on messages conveyed via the mass media, addressing the advantages of small family size and family planning. Mass media can present a wider range of knowledge and lead to adopting contraception. Furthermore, long-running programs focusing on increasing literacy status and wealth status are essential to improve the reproductive health status of women. Similarly, the relation between fertility and child mortality experienced by mothers was found to be very strong and positive in the study. Programs that focus on reduction of infant and child mortality could also be considered, which would also help to reduce fertility. In short, it can be concluded that programs should aim to reduce fertility by focusing on all these identified predictors so that fertility as well as infant and maternal mortality and morbidity can be decreased and the overall well-being of the family maintained and enhanced.

## Competing interests

The author declares that they have no competing interests.

## Authors' contributions

RA, Lecturer at Mahendra Ratna Campus, Kathmandu, conducted data analysis, interpretation, and drafted the manuscript.

## Pre-publication history

The pre-publication history for this paper can be accessed here:

http://www.biomedcentral.com/1471-2393/10/19/prepub
